# *SCRIB* Promotes Proliferation and Metastasis by Targeting Hippo/YAP Signalling in Colorectal Cancer

**DOI:** 10.3389/fcell.2021.656359

**Published:** 2021-04-15

**Authors:** Hengyang Shen, Changzhi Huang, Jingyu Wu, Jie Li, Tao Hu, Zhenling Wang, Hongqiang Zhang, Yu Shao, Zan Fu

**Affiliations:** ^1^Department of General Surgery, The First Affiliated Hospital of Nanjing Medical University, Jiangsu Province Hospital, Nanjing, China; ^2^Department of General Surgery, The Affiliated Jiangning Hospital of Nanjing Medical University, Nanjing, China

**Keywords:** colorectal cancer, *SCRIB*, Hippo signaling pathway, tumour invasion and poor prognosis, cell cycle, cell polarity

## Abstract

The complex in which scribble planar cell polarity protein (*SCRIB*) is located is one of the three main polar protein complexes that play an important role in maintaining epithelial polarity and affecting tumour growth. However, the role of *SCRIB* in colorectal cancer (CRC) remains largely unknown. This study used date from The Cancer Genome Atlas (TCGA) and clinical samples to determine the expression of *SCRIB* in CRC and explored its mechanism through bioinformatics analysis and *in vivo* and *in vitro* experiments. In this study, *SCRIB* was found to be highly expressed in CRC patients, and it was often associated with malignant characteristics, such as proliferation, apoptosis, and epithelial-mesenchymal transition (EMT). Furthermore, we found that *SCRIB* may interact with the Hippo signalling pathway and affect the phosphorylation of YAP and its distribution inside and outside of the nucleus. We concluded that increased expression of *SCRIB* is likely to inhibit the Hippo signalling pathway by promoting YAP phosphorylation. This role of *SCRIB* in the progression of CRC provides an important information for the treatment of CRC.

## Introduction

Colorectal cancer (CRC) is the third most common malignant tumour with the third highest incidence and the second highest mortality rate worldwide, and the age of patients at CRC onset is decreasing (Siegel et al., 2020a,b). The increase in the incidence of CRC among Chinese population in the past decade is closely related to the changes in people’s living habits due to rapid economic development; these changes include unhealthy eating, drinking, smoking, lack of exercise and obesity (Zhu et al., 2017). Although the use of surgery and comprehensive treatment has benefited many CRC patients, nearly half of the patients who undergo radical surgery may relapse, and patients who die from CRC liver metastases account for more than 50% of all CRC-related deaths (Enomoto and Igaki, 2011; Beckers et al., 2018). The occurrence of CRC is an extremely complicated process that may involve biological behaviours such as cell proliferation, metabolism, apoptosis, invasion and metastasis (Hanahan and Weinberg, 2000, 2011). However, the specific mechanisms underlying the occurrence and distant metastasis of CRC have yet to be determined.

The development of solid tumours is actually an process driven by internal and external factors that causes normal cells to proliferate in an unregulated manner and to acquire migratory and invasive capabilities (Hanahan and Weinberg, 2011). In particular, disordered cell polarity is one of the important intracellular factors that is important for the malignant progression of CRC and other epithelial cell tumours (Rothenberg et al., 2010; St Johnston and Ahringer, 2010; Muthuswamy and Xue, 2012; Chatterjee and McCaffrey, 2014). Scribble complexes, which are one of the three types of polar complexes, have a profound impact on the apical–basal polarity of epithelial cells (Assémat et al., 2008; Lee and Vasioukhin, 2008). In several studies, the *SCRIB* gene, which encodes the Scribble protein, is even considered to be a regulator of tumour development and metastasis due to its role in tumour-related mechanisms (Zen et al., 2009; Royer and Lu, 2011). However, considering that the function of *SCRIB* in different, even opposite, in different tumours, it cannot be generally considered to be a cancer-promoting or cancer-suppressing gene (Zhan et al., 2008; Feigin et al., 2014). Our previous study found that there is a polymorphic site in *SCRIB*, namely rs13251492 (*P* = 7.76 × 10^–5^), that is significantly associated with CRC, and we proved that this genetic variation can affect the expression of *SCRIB* (Shen et al., 2020). However, the impact of *SCRIB* on the progression of CRC and its underlying mechanism are not yet known.

Our study determined that *SCRIB* is highly expressed in CRC and indicates a poor prognosis. We performed gain-of-function and loss-of function experiments and found that *SCRIB* promotes the proliferation, invasion and migration of CRC, and inhibits its apoptosis *in vitro* and *in vivo*. The bioinformatics prediction and the corresponding verification experiments together yielded the result that *SCRIB* progression of CRC by influencing the Hippo signalling pathway. Therefore, our study elucidates the mechanism by which *SCRIB* participates in CRC and may provide a new target for the treatment of CRC and a new perspective for clinical decision-making.

## Materials and Methods

### Clinical Samples and Ethical Approval

Human CRC and adjacent normal mucosa samples were obtained from CRC patients who underwent surgery at the First Affiliated Hospital of Nanjing Medical University between 2014 and 2018. Within 5 min of surgical resection, the samples were immediately embedded in the paraffin or transferred to the −80°C freezer. All clinicopathological diagnoses were confirmed by at least two pathologists according to the guidelines of the American Joint Committee on Cancer. All experiments were approved by the Ethics Committee of the First Affiliated Hospital of Nanjing Medical University, and informed consent was obtained from the all the patients before enrolment in this study.

### Cell Culture and Transfections

Normal epithelial colon cells (NCM460 cells) and human CRC cells, including Lovo, HCT116, SW620, DLD-1 and HT-29 cells, were obtained from American Type Culture Collection. RPMI-1640 medium and McCoy’5A medium (HyClone, Logan, UT, United States) were mixed with 10% fetal bovine serum to culture the DLD-1 and HCT116 cells, respectively. The cells were cultured in a humid incubator, which always maintained a temperature of 37°C and an atmosphere of 5% CO_2_. The siRNAs targeting *SCRIB* and the corresponding negative controls (si-NC) were synthesized by RiboBio (Guangzhou, China). The siRNA target sequence (GAAGCAGCTATCCATCCTA) with the highest efficiency for knocking down the expression of *SCRIB* was cloned into the lentivirus vector pGLV3 to by GeneChem (Shanghai, China). GeneChem Company designed and constructed the *SCRIB* overexpression plasmid for our study. Lipofectamine 3000 (Invitrogen) was used for the transfection of cells.

### RNA Extraction and Polymerase Chain Reaction (PCR)

Total RNA was extracted from CRC tissue and CRC cell lines with TRIzol reagent according to the manufacturer’s instructions (Qiagen, Hilden, Germany). The HiScript^®^ III RT SuperMix (Vazyme, Nanjing, China) was used to reverse transcribe the extracted total RNA into cDNA by reverse transcription PCR (RT-PCR). The genes were amplified and their expression levels were detected by quantitative real-time PCR (qRT-PCR) using ChamQ SYBR qPCR Master Mix (Vazyme, Nanjing, China). The results were normalized to the expression levels of *GAPDH*. The primers for the target mRNAs and internal control were as follows: *SCRIB*, forward primer, 5′-CCTCTGTCAAGGGAGTGTCG-3′ and reverse primer, 5′-CCCGAGAGATGAATATGCCCTC-3′; YAP, forward primer, 5′-TAGCCCTGCGTAGCCAGTTA-3′, reverse primer, 5′TCATGCTTAGTCCACTGTCTGT-3′; GAPDH, forward primer, 5′-GGAGCGAGATCCCTCCAAAAT-3′, reverse primer, 5′-GGCTGTTGTCATACTTCTCATGG-3′; ZO-1, forward primer, CGGTCCTCTGAGCCTGTAAG, reverse primer, GGAT CTACATGCGACGACAA; E-cadherin, forward primer, 5′-AC CAGAATAAAGACCAAGTGACCA-3′, reverse primer, 5′-AGCAAGAGCAGCAGAATCAGAAT-3′; N-cadherin, forward primer, 5′-GGACAGTTCCTGAGGGAT CA-3′, reverse primer, 5′-GGATTGCCTTCCATGTCTG T-3′; *Vimentin*, forward primer, 5′-GATGCGTGAGATGGAAGAGA-3′, reverse primer, 5′-GGCCATGTTAACATTGAGCA-3′.

### Western Blot Analysis

Cells and tissue fragments were sonicated and then RIPA lysis buffer (Beyotime, Shanghai, China) was used for protein extraction. The concentrations of the purified proteins were detected by bicinchoninic acid (BCA) protein assay (Beyotime, Shanghai, China). The protein samples were separated by 10% gels using SDS-PAGE and transferred to PVDF membranes (Millipore, Billerica, MA, United States). The antibodies against *SCRIB*, E-cadherin, ZO-1, N-cadherin, Vimentin, Histone H3, Ki67, Cdk4, GAPDH and β-actin were purchased from Abcam (Cambridge, MA, United States). The antibodies against YAP and phospho-YAP (Ser127) were purchased from Cell Signaling Technology (Danvers, MA, United States).

### Scratch Wound Healing Assay and Transwell Assay

The cells were seeded in a 6-well plate at a density of 4×10^5^ cells/well and cultured until the cells covered almost the entire well. A 200-μL pipette tip was used to scrape a linear wound. The culture medium in the dish was replaced with serum-free medium at 0 and 24 hours after injury, and the migrating cells at the edge of the wound were monitored by an inverted microscope. According to the manufacturer’s instructions, Millicell cell culture inserts (24-well inserts with 8-μm pores) were used for Transwell and invasion analyses. To perform the migration assays, the bottom of the insert was seeded with 4 × 10^4^ cells (per well) in 200 μL of serum-free medium, and then, 500 μl of medium containing 10% FBS was added to the lower chamber. For the invasion assay, Matrigel (BD Biosciences, Franklin Lakes, NJ, United States) was added to the insert, and then, 8 × 10^4^ cells (per well) were plated in serum-free medium. Add Medium containing 10% FBS was added to the lower chamber. After 24 hours of incubation, the cells on the bottom of the membrane were fixed and stained with 0.5% crystal violet solution. We used light microscopy to observe the migrating and invading cells and randomly selected 3 fields for counting.

### Cell Proliferation Assay

For the colony formation assay, cells were plated in 6-well plates. We counted the number of colonies 14 days later and photographed representative wells. In addition, a cell proliferation assay was also performed with the 5-ethynyl-2′-deoxyuridine assay (EDU) Kit (Beyotime, Shanghai, China) and Cell Counting Kit 8 (CCK-8) assay (Beyotime, Shanghai, China) according to the manufacturer’s protocols. The detailed methods were described in our previous research (Hu et al., 2020).

### Immunohistochemistry

Immunohistochemistry (IHC) was conducted as previously described (Hu et al., 2020). Tissue sections with a thickness of 4 μm were first deparaffinized with xylene and then rehydrated with gradient alcohol. The samples were incubated with 3% H_2_O_2_ for 10 min at room temperature to block the endogenous peroxidase activity. Finally, the primary antibodies were combined with the proteins in the sample by using the streptavidin peroxidase-conjugated method. Photographs of the sections were obtained using a digital microscope camera. The staining score was used to indicate the expression level of genes, and the specific scoring rules can refer to previous studies.

### Immunofluorescence (IF)

The transfected cells were fixed with 4% paraformaldehyde for 20 min. The cells were incubated in 0.1% Triton X-100 for 4 hours at room temperature to achieve permeabilization. The cells were blocked in the blocking solution for 60 min and gently shaken on a shaker. The blocking solution was removed by washing with phosphate-buffered saline (PBS), and the samples were incubated with diluted primary antibodies against YAP (1:250; Abcam) for 60 min. The primary antibodies were removed, and the cells were washed 3–5 times with PBS for 3–5 min each time. After removing the washing solution, 1 ml of Alexa Fluor488-conjugated secondary antibodies (Beyotime, Shanghai, China) was added and incubated for 60 min in the dark. Fluorescence confocal images was captured by confocal fluorescence microscopy (Zeiss Germany, Germany).

### Animal Experiments

We chose to use 6- to 8-week-old BALB/c nude male mice for the subcutaneous tumour formation experiments. For the tumorigenicity studies, The HCT116 cells stably transfected with lentivirus and DLD-1 cells transfected with the overexpression plasmid were used. A total of 5 × 10^6^ of the indicated cells, including control cells, were subcutaneously injected into the left and right axilla of each nude mouse. The volume and weight of the tumours were measured every 5 days. After 4–6 weeks, xenograft tumours were dissected from sacrificed nude mice and used for IHC. All the animal experiments were approved by the Committee on the Ethics of Animal Experiments of Nanjing Medical University.

### Cell Cycle and Apoptosis Analysis

For cell cycle analysis, we use the appropriate methods to process the cells and set up a negative control group. A total of 1–10×10^5^ cells were washed with PBS and centrifuged at 2,000 rpm per minute. Then, 500 μl of cold 70% ethanol was added to the precipitate for fixation, and the samples were incubated overnight at 4°C. Before staining, we washed away the fixative with PBS, added 100 μl RNase A (KeyGene, Jiangsu, China) and then incubated the samples at 37°C for 30 min. Then, 400 μl PI was added to the tubes, and the samples were incubated at 4°C in the dark for 30 min. Finally, flow cytometry analysis was performed according to the protocol. The apoptosis assay was performed using an Annexin V-FITC/PI (MultiScience, Zhejiang, China). The cells were digested and centrifuged at 1,500 rpm for 5 min. The cells were centrifuged and washed with prechilled PBS. Approximately 1–10×10^5^ cells were collected, including the cells in the culture supernatant. Five hundred microlitres of the working solution diluted in double-distilled water was to resuspend the cells. A total of 5 μl Annexin V-FITC and 10 μl PI were added to each tube. After gentle vertexing, the cells were incubated for 5 min at room temperature in the dark. Finally, flow cytometry analysis was performed according to the protocol.

### TUNEL Staining

According to the manufacturer’s protocol, a one-step TUNEL (TdT-mediated dUTP gap end labelling) apoptosis detection kit (Beyotime, C1090) was used to examine the apoptosis of HCT116 and DLD-1 cells after the knockdown and overexpression of *SCRIB*, respectively. The cells were photographed under an Olympus FSX100 microscope (Tokyo, Japan).

### Statistical Analysis

All the experiments were carried out at least three independent times. All the statistical analyses were performed using Social Sciences 22.0 software (SPSS, CA, United States), and the statistical results were visualized using GraphPad Prism 8.0 (GraphPad Software, CA, United States). The *t*-test was used to compare the differences between the two groups of data that conformed to the Gaussian distribution, while the Wilcoxon test and the Mann-Whitney test were used for the paired and unpaired data with a the non-Gaussian distribution. One-way ANOVA was used to compare the differences between multiple groups of data, and the χ^2^ test was used to compare categorical variables. All the data are presented as the mean ± SD (standard deviation) of triplicates. *P* values <0.05, as determined using two-sided tests, were considered statistically significant.

## Results

### Upregulation of *SCRIB* in CRC Predicts a Poor Prognosis

**FIGURE 1:**
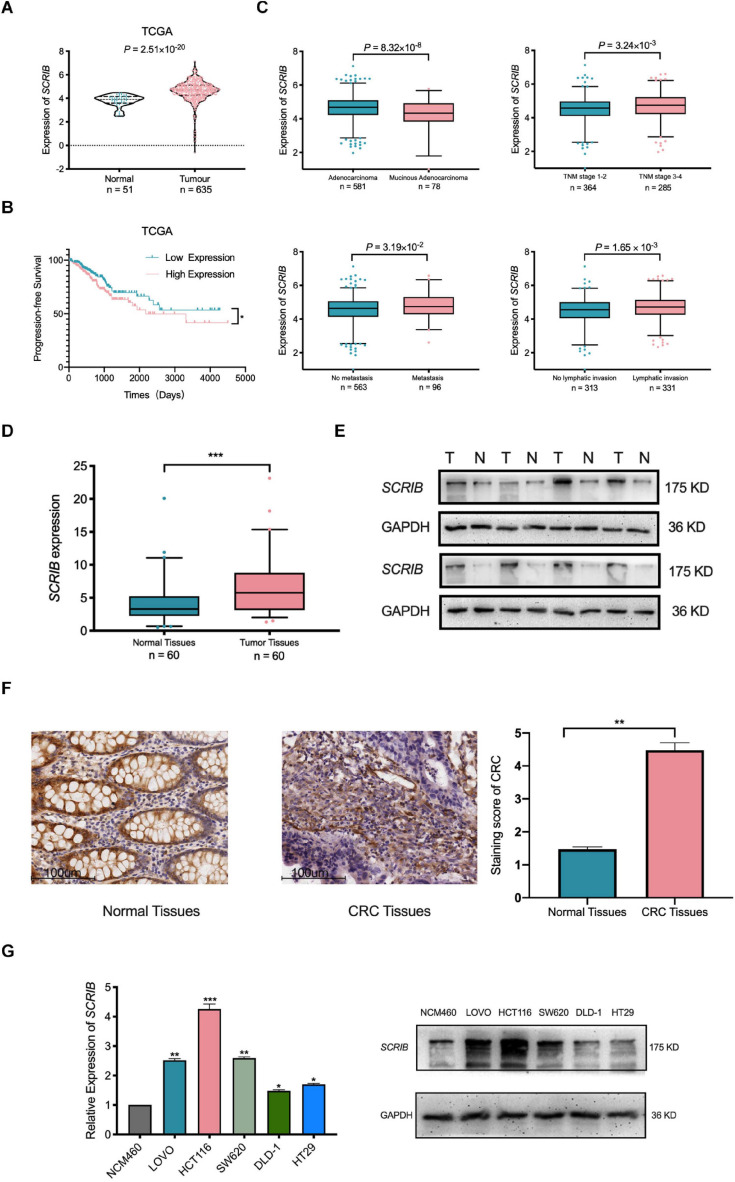
Upregulation of *SCRIB* predicts poor prognosis for patients with colorectal cancer. **(A)** The expression level of *SCRIB* in normal tissues (*n* = 51) and CRC tissues (*n* = 635) from The Cancer Genome Atlas (TCGA) database. **(B)** CRC patients (*n* = 499) with progression-free survival data in the TCGA database were included in the survival analysis. The population was divided into two groups according to the median expression level of *SCRIB*. **(C)** The stratified analysis of CRC patients was based on pathological type, stage, metastasis, and lymphatic invasion. The data were derived from TCGA. Due to the lack of patient data, the total number of stratified analyses is not consistent. **(D)** *SCRIB* expression in 60 paired CRC tissues and adjacent normal tissues. **(E)** The expression of *SCRIB* in eight pairs of CRC tissues (T) and adjacent normal tissues (N) was detected by western blot. **(F)** The expression of *SCRIB* in CRC tissue was assessed by immunohistochemical staining. Representative micrographs show high-level and low-level expression of *SCRIB* in tumour tissues and adjacent nontumorous tissues. Scale bar, 100 μm. **(G)** qRT-PCR showed the expression level of *SCRIB* in NCM460, LOVO, HCT116, SW620, DLD-1, and HT29 cells; western blot showed the protein level of *SCRIB* in these cell lines. *p < 0.05, **p < 0.01, and ***p < 0.001.

In our previous research, a large amount of data from TCGA and the Gene Expression Omnibus database was analysed to illustrate the relationship between *SCRIB* and CRC (Shen et al., 2020). Considering the availability of updated data, we repeated some of these significant analyses. Our new analysis showed that the mRNA level of *SCRIB* in tumour tissues was significantly increased compared to that in normal colorectal tissues (*P* = 2.51×10^−20^) (Figure 1A). In addition, the survival analysis results showed that CRC patients with high *SCRIB* expression were more likely to relapse (Figure 1B). In the stratified analysis of tumour tissue, we found that the different pathological types of CRC, adenocarcinoma and mucinous adenocarcinoma, had obvious differences (*P* = 8.32 × 10^–8^). The expression level of *SCRIB* was higher in advanced patients with tumour-node-metastasis (TNM) staging, and high *SCRIB* expression was also observed in the CRC cancer tissue of patients with metastasis and lymphatic invasion (Figure 1C). Together, these results indicate that the expression of *SCRIB* may have potential value for predicting the progression of CRC. Therefore, we further detected the expression level of *SCRIB* in CRC tissues and analysed the relationship between its expression level and the epidemiological and pathological characteristics of CRC patients (Table 1). The expression of *SCRIB* did not change due to the age, sex, and living habits of CRC patients, but there were indeed differences in patients with different TNM stages (*P* = 0.018), this observation is consistent with the TCGA analysis results. The qRT-PCR and western blot results showed that *SCRIB* substantially upregulated in tumours tissues compared with the adjacent nontumorous tissues at both the mRNA and protein levels (Figures 1D,E). The high expression of *SCRIB* in CRC tissues was also shown by IHC staining of CRC tissues (Figure 1F). The patients were divided into two groups based on high *SCRIB* expression and low *SCRIB* expression to study the relationship between the expression level of *SCRIB* and the clinical characteristics of CRC patients (Table 1). In addition, the expression of *SCRIB* in various CRC cells was higher than that in normal colorectal cells (Figure 1G).

**TABLE 1:** Relevance analysis of *SCRIB* expression in CRC patients.

**Variables**	**All patients**	***SCRIB* Level**		**χ^2^**	***P*^a^**
		**Low**	**HIGH**		
All Cases	60	30	30		
Age (years)				0.317	0.573
<60	18	10	8		
≥60	42	20	22		
Gender				0.278	0.598
Male	36	17	19		
Female	24	13	11		
Smoking status				0.278	0.598
Never	36	19	17		
Ever	24	11	13		
Drinking status				1.27	0.26
Never	42	23	19		
Ever	18	7	11		
Tumor location				0.067	0.796
Colon	29	15	14		
Rectum	31	15	16		
Tumor grade				1.002	0.317
Well+Moderate	49	26	23		
Poor	11	4	7		
TNM staging system				2.443	0.018*
Stage I +II	35	22	13		
Stage III +IV	25	8	17		

^*a*^Chi-square test was used for comparing groups between low and high *SCRIB* expression. *P < 0.05.

### *SCRIB* Upregulation Enhanced the Metastatic Capacity of CRC

**FIGURE 2:**
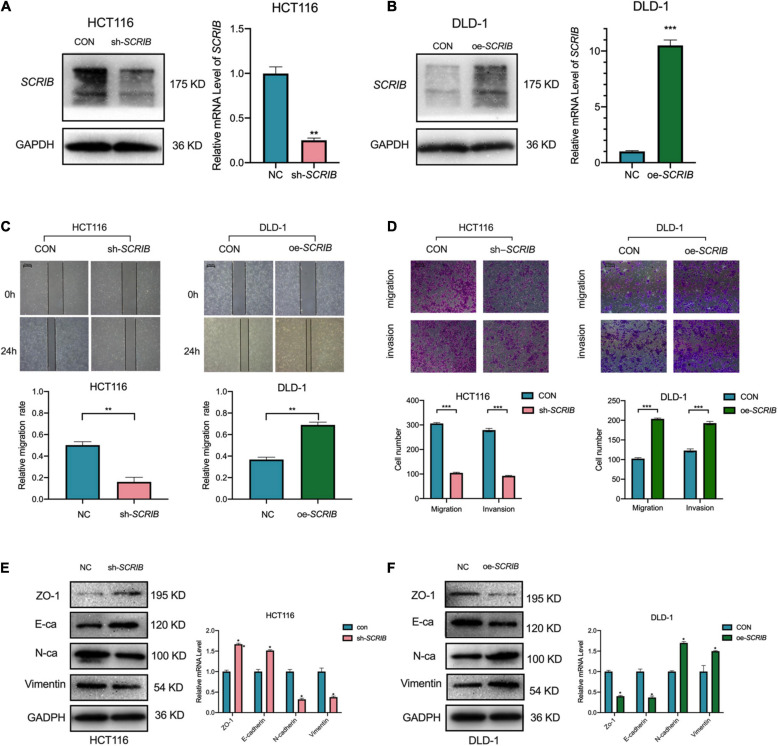
*SCRIB* promotes the migration and invasion of CRC cells *in vitro*. *SCRIB* was knocked down and overexpressed in HCT116 and DLD-1 cells, respectively. The efficiency of knockdown and overexpression was verified by qRT-PCR and WB **(A,B)**. **(C)** Effects of *SCRIB* on migration in CRC cell lines were detected by wound healing assay, scale bars: 100 μm. **(D)** Cell migration and invasion of HCT116 and DLD-1 cells as measured by Transwell assays; scale bars: 100 μm. **(E)** Western blot analysis of the tight junction protein ZO-1, epithelial marker E-cadherin, and mesenchymal markers N-cadherin and Vimentin after *SCRIB* knockdown in HCT116 cells. **(F)** The same molecules were observed in DLD-1 cells overexpressing *SCRIB*. The data represent the mean ± SD from three independent experiments. Student’s *t*-test was used to determine statistical significance: *P < 0.05, **P < 0.01, and ***P < 0.001.

Based on the differential expression of *SCRIB* in different CRC cells, we performed loss-and gain-of-function assays with HCT116 cells, which have high *SCRIB* expression, and DLD-1 cells, which have low *SCRIB* expression (Figure 1G). First, the knockdown lentivirus and overexpression plasmids were transfected into HCT116 and DLD-1 cells, respectively. The knockdown and overexpression efficiencies were examined by qRT-PCR and western blot respectively (Figures 2A,B). Considering the distribution of *SCRIB* expression in patients with different TNM stages, distant metastasis and lymphatic invasion (Figure 1C), we have a strong interest in studying the impact of *SCRIB* on the invasion and metastasis of CRC cell lines. The wound healing assays demonstrated that *SCRIB* downregulated inhibited the migration of HCT116 cells and *SCRIB* overexpression significantly increased the migration of DLD-1 cells compared to that of control cells (Figure 2C). Transwell assays showed that downregulated *SCRIB* reduced the migration and invasion of HCT116 cells relative to those of control cells. Correspondingly, DLD-1 cells overexpressing *SCRIB* showed enhanced cell migration and invasion capabilities (Figure 2D). We used qRT-PCR and western blotting to detect the expression levels of several epithelial-mesenchymal transitions (EMT) markers to further verify whether *SCRIB* promotes CRC transfer by regulating EMT. ZO-1 is a tight junction protein, E-cadherin is an epithelial marker, and N-cadherin and vimentin are mesenchymal markers. The results of Figure 2E show that the knockdown of *SCRIB* in HCT116 cells increased the expression of ZO-1 and E-cadherin and decreased the expression of the N-cadherin and vimentin at the mRNA and protein levels (Figure 2E). In contrast, DLD-1 cells overexpressing *SCRIB* showed a significant decrease in the expression of E-cadherin and ZO1 and an increased in the expression of vimentin and N-cadherin (Figure 2F).

### The Expression of *SCRIB* Affects the Proliferation and Cell Cycle of CRC Cells

**FIGURE 3:**
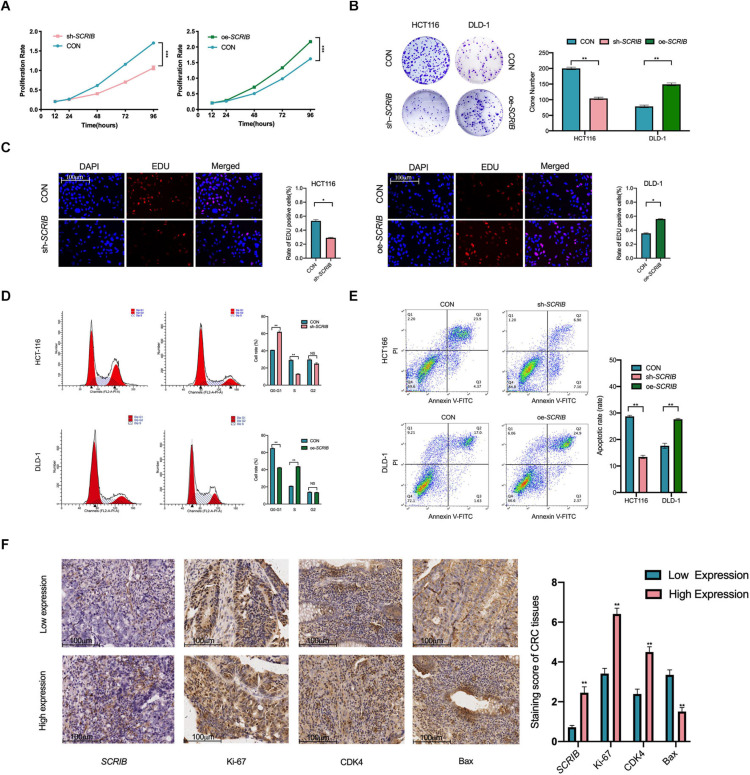
*SCRIB* promotes CRC cell proliferation and inhibits apoptosis *in vitro.* **(A)** Cells were seeded in 96-well plates after transfection with oe-*SCRIB* and sh-*SCRIB* and the corresponding empty vectors, and the cell number was assessed on days 1–4 using CCK-8 assays. The knockdown of *SCRIB* suppressed the proliferation of HCT116 cells, whereas the overexpression of *SCRIB* promoted the proliferation of DLD-1 cells. **(B)** Effect of overexpression or knockdown of *SCRIB* on the colony formation ability of CRC cells; representative graphs are shown. **(C)** EdU immunofluorescence staining confirmed the function of *SCRIB* in the proliferation of CRC cells, scale bars: 100 μm. **(D)** Flow cytometry showing significant increases or decreases in the proportion of HCT116 cells in the G1 or S phase, respectively, when *SCRIB* was knocked down. In the *SCRIB*-overexpressing DLD-1 cells, the number of cells in the G1 or S phase decreased or increased, respectively. **(E)** The apoptotic rates (LR + UR) of transfected cells were detected by flow cytometry (Q2 + Q3). LR, early apoptotic cells; UR, terminal apoptotic cells. **(F)** Immunohistochemical staining of Ki-67, CDK4 and Bax was performed, and their expression was compared between *SCRIB* high-expressing CRC tissues and *SCRIB* low-expressing tissues, scale bars: 100 μm. The data are shown as the mean ± SD of three independent experiments, *P < 0.05, ** *P* < 0.01, and ***P < 0.001.

We further studied the role of *SCRIB* in CRC cell proliferation and cell cycle progression. First, we used a CCK-8 assay to study the effect of *SCRIB* on the proliferation of CRC cells by observing the absorbance of the cells for 5 consecutive days. The results of the CCK-8 experiment showed that the proliferation ability of HCT116 cells with downregulated *SCRIB* was reduced, while the upregulation of *SCRIB* enhanced the proliferation ability of DLD-1 cells (Figure 3A). Colony formation assays showed that HCT116 cells with *SCRIB* knocked down formed fewer colonies than control cells, while DLD-1 cells overexpressing *SCRIB* formed more colonies than control cells (Figure 3B). The results of the EDU assays further confirmed that *SCRIB* can promote cell proliferation (Figure 3C). In light of the function of *SCRIB* in CRC cell proliferation, we considered whether its expression level would cause differences in the cell cycle distribution and apoptosis of CRC cells. An increased number of cells in the G0 and G1 phases and a decreased number of cells in the S phases were observed in the HCT116 cells with *SCRIB*-knocked down compared with control cells. In contrast, the overexpression of *SCRIB* promoted the G1-to-S phase transition in DLD-1 cells (Figure 3D). In addition, transfected HCT116 cells exhibited a reduced proportion of apoptotic cells compared to the control cell. The proportion of apoptotic cells in DLD-1 cells overexpressing *SCRIB* increased significantly (Figure 3E). Based on the results of the above experiments, we further examined the effects of changes in *SCRIB* expression on cell proliferation and cell cycle progression at the tissue level. In immunohistochemical (IHC) staining, the expression levels of Ki67, CDK4 and Bax were used as markers to detect cell proliferation, cell cycle progression and apoptosis. We observed that *SCRIB* expression was positively correlated with Ki67, CDK4 and Bax expression at the tissue level (Figure 3F).

### *SCRIB* Contributes to Tumour Proliferation and Progression *in vivo*

**FIGURE 4:**
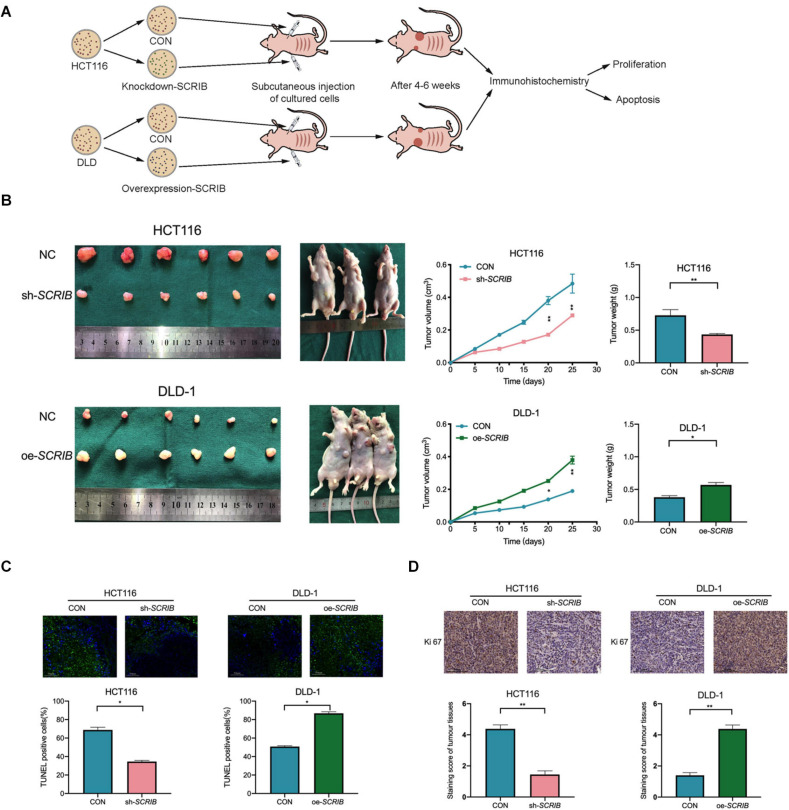
*SCRIB* enhances tumour growth *in vivo*. **(A)** Nude mice were subcutaneously injected with HCT116 cells transfected with lentivirus and DLD-1 cells transfected with overexpression plasmid and their corresponding control cells. The established tumours in the right and left groins of the mice were measured every 5 days, and the mice were sacrificed 4–6 weeks after implantation. **(B)** Knockdown of *SCRIB* inhibited the tumour growth of HCT116 cells in nude mice, while overexpression of *SCRIB* promoted the tumour growth of DLD-1 cells in nude mice. **(C)** Apoptotic cells in tumour tissue were detected by TUNEL assay, and the number of TUNEL-positive cells was quantified; scale bars: 50 μm. **(D)** Protein levels of Ki67 in the tumour samples were determined by IHC, scale bars: The data are shown as the mean ± SD of three independent experiments, *P < 0.05, **P < 0.01.

As shown in the model diagram, HCT116 cells with *SCRIB* knocked down, DLD-1 cells overexpressing *SCRIB*, and the corresponding control cells were injected subcutaneously into nude mice (Figure 4A). Tumour growth was markably suppressed by the knockdown of *SCRIB* as shown by lower final tumour volumes and weights than those of the control tumours, and overexpression of *SCRIB* promoted tumour growth (Figure 4B). A TUNEL assay was performed, and the results showed that the TUNEL-positive rate in tumour tissues with relatively high *SCRIB* expression was significantly higher than that in tumour tissues with relatively low *SCRIB* expression (Figure 4C). Furthermore, we performed immunohistochemical staining of the tumour tissues in nude mice. A decrease in the expression of Ki67 in *SCRIB* knockdown tumours was observed by IHC staining (Figure 4D).

### *SCRIB* Promotes the Activation of the Hippo/YAP Pathway in CRC Cells

**FIGURE 5:**
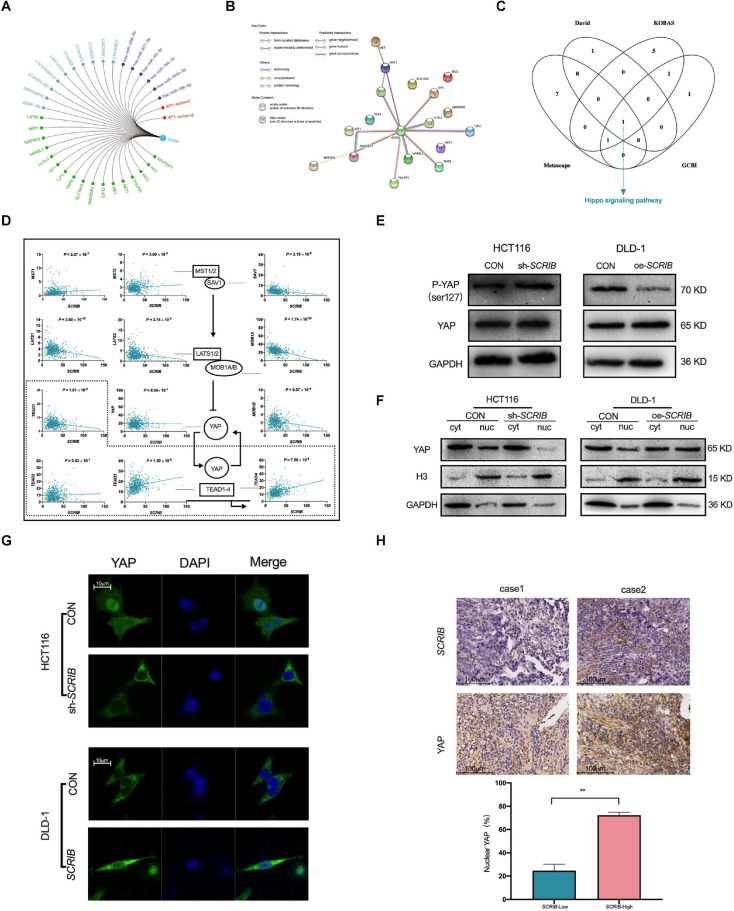
| *SCRIB* promotes the Hippo/YAP signalling pathway in CRC cells. **(A)** *SCRIB*-related regulatory network. Green represents interacting proteins, light blue represents related lncRNAs, purple represents targeted miRNAs, and red represents activated transcription factors. **(B)** Interactions between proteins predicted by the STRING database. The empty nodes represent the 3D structure position of the protein, and the filled nodes represent the known or predicted 3D structure of the protein. Proteins determined to be related to each other are connected by light blue and dark purple, and these relations have been verified by databases and experiments, respectively. Among the predicted protein interactions, dark green represents the gene neighbourhood, red represents gene fusions, and dark blue represents gene cooccurrence. The remaining lines represent weaker connections between proteins: light green represents text mining, black represents coexpression, and light purple represents protein homology. **(C)** David, KOBAS, Metascape and GCBI were used to predict the downstream pathways of *SCRIB*, and the Hippo signalling pathway was the only signalling pathway identified in all four databases. **(D)** The correlation between *SCRIB* and the core genes of the Hippo signalling pathway was analysed through the TCGA database. The dashed box indicates the nucleus. A straight arrow indicates activation, and a straight blunt arrow indicates inhibition. **(E)** Nuclear and cytoplasmic protein separation from the indicated CRC cells was performed to detect the expression of YAP by western blotting. Histone H3 and GAPDH were used as the loading controls for nuclear proteins and cytoplasmic proteins, respectively. **(F)** Cell lysates from the indicated cells were subjected to immunoblotting for phosphorylated YAP (Ser127) and total YAP. **(G)** Representative images showing the influence of different expression levels of *SCRIB* on the intracellular distribution of YAP; scale bar: 10 μm. **(H)** Representative images of immunohistochemical staining for *SCRIB* and YAP in sections from CRC tumours, scale bar: 100 μm. **P < 0.01.

To further study the mechanism by which *SCRIB* mediates tumour biological behaviour, we summarized the *SCRIB*-related molecules, including miRNAs, lncRNAs, transcription factors and interacting proteins, reported in the literature (Figure 5A and oSupplementary Table 1). We focused on the proteins related to *SCRIB* and used the String database to predict the relationships between *SCRIB* and these proteins. We clearly observed that the protein encoded by *SCRIB* interacts with most proteins, including those that have been verified by experiments and predicted by bioinformatics (Figure 5B). Then, we analysed the pathway enrichment of these proteins through DAVID, KOBAS, Metascape and GCBI to identify the signalling pathways targeted by *SCRIB*. Ultimately, the Hippo signalling pathway was revealed to be the only signalling pathway enriched in all four databases (Figure 5C and Supplementary Table 2). CRC data from the TCGA database were used to analyse the correlation between the core genes and downstream target genes of the Hippo signalling pathway and *SCRIB* (Figure 5D). We first observed that there was no correlation between the expression of *SCRIB* and YAP, a key gene of the Hippo signalling pathway. However, the target genes TEAD3 and TEAD4, which are activated by YAP in the nucleus, were significantly positively correlated with *SCRIB* (*P* = 1.50 × 10^–8^ and *P* = 7.50 × 10^–8^, respectively). Although the expression levels of MST1/2 and SAV1 were not obviously altered as the expression levels of *SCRIB* increased, LAST1/2 and MOB1A/B were all negatively correlated with *SCRIB*. The activation of the upstream genes of the Hippo signalling pathway, such as MST1/2 and LATS1/2, causes serine 127 of YAP to be phosphorylated, which traps YAP in the cytoplasm to promote transcription (Zhao et al., 2007). Therefore, we assume that the increase in the expression of *SCRIB* does not directly affect the expression of YAP but rather prevents the LAST1/2 and MOB1A/B complexes from reducing the phosphorylation of YAP, thereby indirectly causing more YAP to enter the nucleus to activate the transcription of the downstream TEAD family of genes. We found by western blot that when *SCRIB* was knocked down or overexpressed, the protein level of YAP did not change significantly, but the level of phosphorylated YAP (p-YAP) increased or decreased accordingly (Figure 5E). In addition, the cytoplasm/nuclear protein extraction assay showed that the nuclear accumulation of YAP in *SCRIB*-knockdown HCT116 cells was lower than that in control cells, while the nuclear accumulation of YAP in *SCRIB-*overexpressing DLD-1 cells was greater than that in control cells (Figure 5F). The IF assay showed that YAP in HCT116 cells mainly accumulates in the nucleus, but the knockdown of *SCRIB* maintains YAP in the cytoplasm. The overexpression of *SCRIB* in DLD-1 cells significantly increased the level of YAP in the nucleus (Figure 5G). IHC of the CRC specimens revealed a positive correlation between *SCRIB* expression and YAP nuclear localization (Figure 5H).

### Agonists and Inhibitors of the Hippo Signalling Pathway Can Restore *SCRIB*-Mediated CRC Cell Proliferation, Invasion and Migration

**FIGURE 6:**
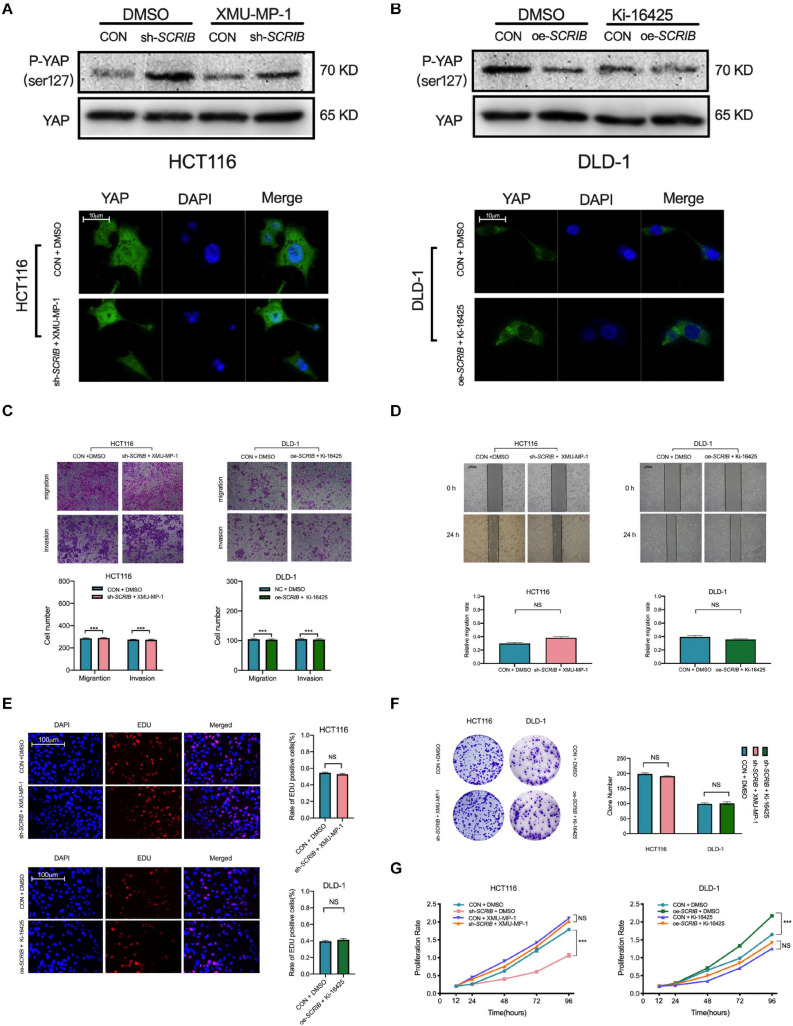
Agonists and inhibitors of the Hippo signalling pathway can restore *SCRIB*-mediated CRC cell proliferation, invasion and migration. Hippo signalling pathway agonists and inhibitors were used to treat the indicated CRC cells, and the corresponding control cells were treated with DMSO to eliminate errors caused by the drugs. **(A)** Western blotting was used to detect the phosphorylated YAP (Ser127) and total YAP levels after the cells were treated. **(B)** Representative images and quantification of immunofluorescence staining for YAP distribution after cells were treated with XMU-MP-1 (1 μM) and Ki-16425 (2 μM) for 12 h. **(C,D)** Cell invasion and migration were detected by Transwell assay and scratch wound healing assay at various time points (ns: no significance). **(E–G)** Cell proliferation was detected by EdU, colony formation and CCK-8 assays at various time points (***P < 0.001, ns: no significance).

XMU-MP-1 is a reversible and selective MST1/2 inhibitor that promotes YAP translocation to the nucleus by reducing the phosphorylation of YAP (Fan et al., 2016). Ki16425 inhibits the dephosphorylation of YAP induced by serum-borne lysophosphatidic acid (LPA) and thus reduces the amount of YAP in the nucleus (Yu et al., 2012). We used these two drugs to determine that the effect of *SCRIB* on the malignant phenotype of CRC cells is dependent on YAP. To a certain extent, the use of these drugs reversed the phosphorylation status of YAP, which changes with the expression of *SCRIB* (Figure 6A). An immunofluorescence experiment also showed that the accumulation of YAP in the cytoplasm was no longer changed due to the use of pathway agonists and inhibitors (Figure 6B). In addition, drug treatment eliminated the *SCRIB*-mediated increase in cell proliferation, invasion and migration (Figures 6C–G). In summary, these results suggest that YAP is essential for the *SCRIB*-mediated malignant phenotype of CRC cells.

## Discussion

In this study, we focused our work on the *SCRIB*-mediated regulation of CRC progression. Our research started by verifying the expression level of *SCRIB* in CRC tissues, including an analysis of the available data in the TCGA database and verification with samples from our laboratory. We have discussed in our previous works that the trend of *SCRIB* expression in different tumours may be different, even opposite (Shen et al., 2020). Although we previously predicted that *SCRIB* may have a significant impact on the development of CRC, it has not been experimentally confirmed. In this study, we not only determined that *SCRIB* is highly expressed in CRC tissues and promotes the progression of CRC but also further studied the mechanism.

The establishment of cell polarity and intercellular adhesion lead to high-order structural organization, and the disruption or complete loss of this organization is often accompanied by neoplastic transformation (Lee and Vasioukhin, 2008). However, the regulation of cell polarity is extremely complicated. On the apical and basal sides of cells, different polar protein complexes antagonize each other to maintain cell polarity (Bilder, 2004; Margolis and Borg, 2005; Suzuki and Ohno, 2006). A large number of studies have found that the expression of the Scrib-Lgl-Dlg polar protein complex is reduced or completely lost in the primary tumours of human patients (Cavatorta et al., 2004; Navarro et al., 2005; Gardiol et al., 2006; Kuphal et al., 2006). However, the conclusion that the deletion of the cell polarity genes dlg, lgl or scib can cause tumours in *Drosophila* is not completely applicable to mammals. Some studies have shown that the deletion of these genes in mammals may only cause asymmetric cell division and not cancer (Klezovitch et al., 2004; Mahoney et al., 2006; Cuthbert et al., 2007). One of the reasons may be that compared with *Drosophila*, in the mammalian system, the core cell polarity components exhibit large amounts of redundancy, so the mutation of a single gene is not enough to cause tumours. In our study, we concluded that the increased expression of *SCRIB* promotes the progression of CRC by affecting proliferation, invasion, metastasis, and apoptosis. It is worth noting that during our research, we found that with the increase in *SCRIB*, the expression of E-cadherin and ZO-1 was downregulated and the expression of vimentin and N-cadherin, which are key epithelial markers of EMT, was upregulated. In the EMT process, epithelial cells acquire invasive capacities, mainly through the weakening of cell-cell junctions and the rearrangement of the cytoskeleton. However, before that, the first event that occurs in epithelial cells is the loss of apical–basal polarity (Kalluri and Weinberg, 2009; De Craene and Berx, 2013; Lamouille et al., 2014). The study by Jung et al. (2019) explained that apical–basal polarity can directly affect the EMT process to inhibit invasion and metastasis and even serve as a critical checkpoint. Wan et al. (2018) found that *SCRIB* was overexpressed in human liver cancer cells, and the dysregulation of *SCRIB* may play an important role in hepatocarcinogenesis and HCC cell dissemination.

An interaction between *SCRIB* and the Hippo signalling pathway has been reported (Enomoto and Igaki, 2011; Martin-Belmonte and Perez-Moreno, 2011), and this interaction was used as the basis for listing *SCRIB* as a gene related to the Hippo signalling pathway in our previous studies (Shen et al., 2020). In this study, we again identified the Hippo signalling pathway as the pathway that is most closely related to *SCRIB* through bioinformatics analysis. Our work found that YAP, the core molecule of the Hippo signalling pathway, did not change with increasing *SCRIB* expression. However, the expression levels of genes targeted by YAP in the nucleus increased accordingly. Considering that YAP affects downstream target genes by entering the nucleus, we hypothesized that the increased expression of *SCRIB* likely promotes YAP translocation to the nucleus; we subsequently confirmed this hypothesis through experiments. We found that four genes, LATS1/2 and MOB1A/B, which are closely associated with YAP and are upstream of the Hippo signalling pathway, are negatively correlated with *SCRIB* in terms of expression. It is well known that the Hippo signalling pathway functions to phosphorylate YAP through a series of upstream phosphorylation cascades. This process prevents YAP from entering the nucleus and ultimately controls excessive cell growth (Zhao et al., 2007; Martin-Belmonte and Perez-Moreno, 2011). Combining our research results, we can conclude that the high expression of *SCRIB* in CRC tissues downregulates the LATS1/2 and MOB1A/B genes, reducing the phosphorylation of YAP, increasing its translocation to the nucleus, and ultimately activating nuclear oncogenes.

Our research still has some limitations. First, the number of CRC tissue samples used was not large, which makes our correlation analysis of the expression of *SCRIB* and the pathological characteristics of CRC patients have a certain deviation. On the other hand, due to the limitation of the experimental conditions, the specific mechanism by which *SCRIB* affects YAP is not detailed. How genes upstream of the Hippo signalling pathway, such as LATS1/2, participate in the process by which *SCRIB* regulates YAP still requires further exploration.

**FIGURE 7:**
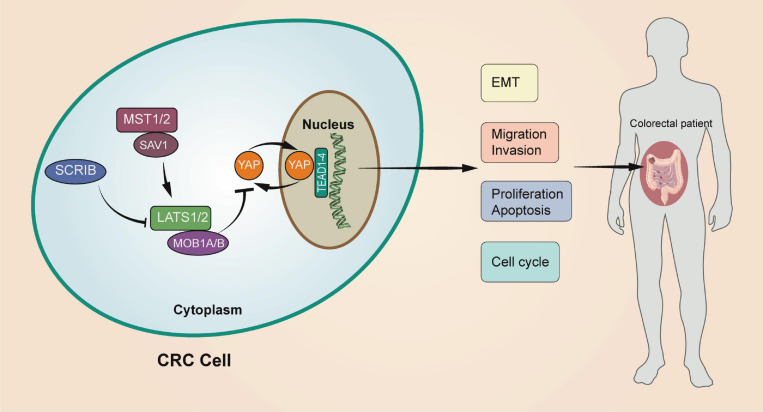
A schematic model of the role of *SCRIB* in regulating CRC. The upregulation of *SCRIB* reduced the expression of LATS1/2 and MOB1A/B to further inhibit the phosphorylation of YAP. Unphosphorylated YAP can translocate to the nucleus to activate transcription of the target genes TEAD1-4. The *SCRIB*-YAP-Hippo axis ultimately affects the disease progression of CRC patients by affecting the proliferation, invasion, migration, and apoptosis of CRC cells.

In conclusion, we verified that *SCRIB* is highly expressed in CRC and participates in tumour progression through *in vivo* and *in vitro* experiments. In addition, we explained that the effect of *SCRIB* on colorectal cancer is achieved by affecting the nuclear translocation of YAP, the core molecule of the Hippo signalling pathway (Figure 7). Our work complements the study of the interaction between polar proteins and the Hippo signalling pathway and provides a possibility of identifying new therapeutic targets for CRC.

## Data Availability Statement

The datasets presented in this study can be found in online repositories. The names of the repository/repositories and accession number(s) can be found in the article/Supplementary Material.

## Ethics Statement

The studies involving human participants were reviewed and approved by Ethics Committee of The First Affiliated Hospital of Nanjing Medical University. The patients/participants provided their written informed consent to participate in this study. The animal study was reviewed and approved by Committee on the Ethics of Animal Experiments of Nanjing Medical University.

## Author Contributions

HS performed molecular biology experiment and write the main manuscript. ZF designed the whole project and supervised all experiments. CH, JW, and JL conducted all experiments and analysed the data. TH, ZW, HZ, and YS provided support with experimental and clinical techniques. All authors read and approved the final manuscript.

## Conflict of Interest

The authors declare that the research was conducted in the absence of any commercial or financial relationships that could be construed as a potential conflict of interest.

## Abbreviations

*SCRIB*: scribble planar cell polarity protein
CRC: colorectal cancer
TCGA: The Cancer Genome Atlas
YAP: Yes1 associated transcriptional regulator
PCR: polymerase chain reaction
qRT-PCR: quantitative real-time polymerase chain reaction
RT-PCR: reverse transcription polymerase chain reaction
EDU: 5-Ethynyl-2’-deoxyuridine
CCK-8: Cell Counting Kit 8
IHC: immunohistochemistry
IF: Immunofluorescence
PBS: phosphate buffer saline
TNM: tumour-node-metastasis
EMT: epithelial-mesenchymal transition
LPA: lysophosphatidic acid
