# Supplementary material for: SCRIB Promotes Proliferation and Metastasis by Targeting Hippo/YAP Signalling in Colorectal Cancer
Source: Front Cell Dev Biol. 2021 Apr 15;9:656359. doi: 10.3389/fcell.2021.656359 (PMC8084105; doi:10.3389/fcell.2021.656359)
Supplement: Supplementary file 1 [file Table_1.docx]

| **Supplement Table 1. Proteins, lncRNAs, miRNAs and transcription factors that have been reported in previous studies that had a regulatory relationship with *SCRIB*.** | | | |
| --- | --- | --- | --- |
| **Protein** | **Ensembl ID** | **Relationship with *SCRIB*** | **PMID** |
| LATS1 | ENSG00000131023 | association | PMID:24362629 |
| MST1 | ENSG00000173531 | association | PMID:24362629 |
| MAP3K3 | ENSG00000198909 | physical association | PMID:14743216 |
| VANGL2 | ENSG00000162738 | complex | [PMID:16791850](https://www.gcbi.com.cn/gclib/html/pubmed/detail/16791850) |
| LLGL2 | ENSG00000073350 | complex | PMID:16791850 |
| GIT1 | ENSG00000108262 | physical association | PMID:15975580 |
| TJP2 | ENSG00000119139 | physical association | PMID:15975580 |
| TRIP6 | ENSG00000087077 | direct interaction | PMID:16137684 |
| SLC15A5 | ENSG00000188991 | physical association | PMID:24550280 |
| ANKRD50 | ENSG00000151458 | physical association | PMID:24550280 |
| DLK2 | ENSG00000171462 | physical association | PMID:24550280 |
| MET | ENSG00000105976 | physical association | PMID:24550280 |
| NET1 | ENSG00000173848 | physical association | PMID:24550280 |
| PHLPP1 | ENSG00000081913 | physical association | PMID:21701506 |
| MCC | ENSG00000171444 | physical association/association | PMID:19555689 |
| APC | ENSG00000134982 | physical association | PMID:16611247 |
| ARHGEF7 | ENSG00000102606 | Physical association/ Association | PMID:21132015、24550280、15975580 |
| **lncRNA** | **PMID** | **miRNA** | **PMID** |
| LINC01667 | PMID:26186194 | hsa-miR-296-5p | PMID:21643016 |
| LINC01561 | PMID:26186194 | hsa-miR-877-3p | PMID:23622248 |
| C1orf220 | PMID:26186194 | hsa-miR-93-3p | PMID:23622248 |
| C17orf102 | PMID:26186194 | hsa-miR-766-3p | PMID:23622248 |
| C17orf82 | PMID:26186194 | hsa-miR-193b-3p | PMID:23622248 |
| LINC00312 | PMID:23602568 | hsa-miR-186-5p | PMID:23622248 |
| LOC101928140 | PMID:25231870 | **Transcription Factor** | **PMID** |
| STARD4-AS1 | PMID:25231870 | WT1-isoform1 | PMID:20571064 |
| MIR193BHG | PMID:25231870 | WT1-isoform2 | PMID:20571064 |
| BDNF-AS | PMID:25231870 |  |  |
| PMID: PubMed Unique Identifier | |  |  |

| **Supplement table 2. The results of pathway enrichment of predicted genes in four databases.** | | |
| --- | --- | --- |
| Database | Pathway_Name | Pathway_ID |
| Metascape |  |  |
|  | SCRIB-GIT1-ARHGEF7 | CORUM |
|  | PID CDC42 REG PATHWAY | M83 |
|  | **Hippo signaling pathway** | hsa04390 |
|  | regulation of protein serine/threonine kinase activity | GO:0071900 |
|  | cell morphogenesis involved in differentiation | GO:0000904 |
|  | Rho protein signal transduction | GO:0007266 |
|  | Tight junction | hsa04530 |
|  | ameboidal-type cell migration | GO:0001667 |
|  | microtubule cytoskeleton organization | GO:0000226 |
| David |  |  |
|  | **Hippo signaling pathway** | hsa04390 |
|  | Regulation of actin cytoskeleton | hsa04810 |
| KOBAS |  |  |
|  | **Hippo signaling pathway** | hsa04390 |
|  | Tight junction | hsa04530 |
|  | Regulation of actin cytoskeletal | hsa04810 |
|  | Epithelial cell signaling | hsa05120 |
|  | Human papillomavirus infection | hsa05165 |
|  | Gastric cancer | hsa05226 |
|  | Wnt signaling pathway | hsa04310 |
|  | Hepatocellular carcinoma | hsa05225 |
| GCBI |  |  |
|  | **Hippo signaling pathway** | hsa04390 |
|  | Tight junction | hsa04530 |
|  | Viral carcinogenesis | hsa05203 |
|  | Human papillomavirus | hsa05165 |
| Metascape: http://metascape.org/gp/index.html#/main/step1 | | |
| David: https://david.ncifcrf.gov/home.jsp | | |
| KOBAS: http://kobas.cbi.pku.edu.cn/kobas3/?t=1 | | |
| GCBI: https://www.gcbi.com.cn/gclib/html/index | | |
